# Correlation of insulin resistance-related indicators and obesity-related indicators with sarcopenic obesity and development of diagnostic models: NHANES 1999–2006

**DOI:** 10.3389/fnut.2024.1492191

**Published:** 2024-11-28

**Authors:** Chen Zhang, Xintao Huang, Hao Lu, Mo Chen, Junyu Wang, Huisheng Deng

**Affiliations:** Department of General Practice, The First Affiliated Hospital of Chongqing Medical University, Chongqing, China

**Keywords:** sarcopenic obesity, diagnostic model, Lasso, NHANES, random forest

## Abstract

This study investigates the correlation between insulin resistance and obesity indicators with sarcopenic obesity (SO) and develops diagnostic models. Utilizing the 1999–2006 National Health and Nutrition Examination Survey (NHANES) database, the research included 5,574 adults. Sarcopenic obesity was defined following the 2022 consensus by ESPEN and EASO. The study analyzed indicators such as the triglyceride-glucose index (TyG), homeostasis model assessment of insulin resistance (HOMA-IR), body roundness index (BRI), and lipid accumulation product (LAP). Results indicated a significant positive correlation between these indicators and SO, with the strongest association observed for TyG-WHtR. Predictor variables were identified through logistic and Lasso regression, including age, sex, weight-height ratio (WHtR), and TyG-WCR. The diagnostic model demonstrated good predictive performance with AUC values of 0.897 for internal validation and 0.853 for external validation. The study underscores the importance of early identification of SO patients and provides a theoretical foundation for future prevention and management strategies. Limitations include the cross-sectional study design and the potential limited generalizability of the model based on the American population.

## 1 Introduction

Sarcopenic obesity (SO), a growing health concern due to the aging global population (1, 2), has a reported prevalence ranging from 13 to 23% (3). Despite varying definitions and diagnostic criteria across studies, SO is consistently recognized as a significant independent risk factor for frailty and mortality, particularly in patients with common comorbidities (4, 5).

In alignment with the 2022 consensus established by the European Society for Clinical Nutrition and Metabolism (ESPEN) and the European Association for the Study of Obesity (EASO), Sarcopenic Obesity is defined as a condition wherein sarcopenia and obesity are not merely coexisting but rather characterized by a reduction in relative skeletal muscle mass (ALM/W) and an elevation in fat mass percentage (FM%) (6, 7). This condition diverges from isolated sarcopenia and obesity, as it is associated with a relatively higher mortality rate and an increased risk of cardiovascular diseases (8–12). Li et al. (13) propose that SO is linked to the onset of chronic diseases, with the severity of sarcopenia correlating with disease progression. This suggests that early detection of SO could be instrumental in preventing or managing chronic diseases.

Studies have shown that the pathogenesis of SO is closely related to insulin resistance (IR) and obesity-related inflammation (14). Obesity is a risk factor for sarcopenia (15–17), so the global obesity epidemic (18) will increase the incidence of SO with age. Although there are similar trends in SO in different regions (19, 20), the prevalence of obesity could potentially influence future incidence rates of SO. Consequently, it becomes imperative to employ IR and obesity indicators as screening tools for SO.

The triglyceride-glucose index (TyG) and related metrics, along with the homeostasis model assessment of insulin resistance (HOMA-IR) are established markers for IR, with TyG showing greater predictive power for metabolic diseases over HOMA-IR (21). Adjusting TyG for WHtR and BMI enhances its predictive value in non-alcoholic fatty liver disease (NAFLD) (22). The novel obesity indicator, body roundness index (BRI) more accurately reflects visceral fat distribution than conventional anthropometrics (23), while lipid accumulation product (LAP), as a cost-effective marker of lipotoxicity, shows promise in NAFLD screening (24). However, the correlation between these indicators and SO, as well as the dose–response relationship, remains unclear, making the research on predictive value and screening capabilities yet to emerge.

In summary, the early identification of SO and the implementation of efficacious interventions to reduce its prevalence are of paramount importance. This is crucial for not only preventing and managing chronic diseases at the individual level and reducing mortality rates but also for preventing a future public health crisis due to the rising prevalence of SO. To date, no diagnostic model for insulin resistance has been developed. Therefore, this study conducted a cross-sectional study using the National Health and Nutrition Examination Survey (NHANES) database with two objectives: the first was to investigate the correlation between IR and obesity-related markers and SO; the second, and modified, was to develop a diagnostic model capable of early identification of SO. The second of these objectives served as the main research aim of this study.

## 2 Materials and methods

### 2.1 Database

The data for this cross-sectional study were obtained from 5,574 adults who participated in the NHANES during the 1999–2006 cycle, and included data including demographic data (age, sex, education, race, marital status, family income-to-poverty ratio, sampling weights), risk behaviors (smoking, alcohol use), history of disease (diabetes, hypertension), anthropometric data (waist circumference calf circumference, arm circumference, weight, height), dual-energy X-rays absorptiometry data (DXA), laboratory test data (LDL-cholesterol, triglycerides, fasting glucose, insulin), physical active data and nutritional survey data (energy intake, protein intake, total fat intake). A flow chart was drawn based on the inclusion process of the above relevant variables (for details, see Supplementary Figure S1) The National Center for Health Statistics (NCHS) research ethics review board approved the NHANES data survey, and written informed consent constituted the inaugural step in the experimental procedure. A comprehensive account of each investigation can be found elsewhere (25).

Anthropometric measurements were obtained utilizing a Toledo electronic scale, Seca electronic rangefinder, and a steel tape measure, and whole-body dual-energy X-ray absorptiometry (DXA) scans were conducted using a Hologic QDR-4500A fan-beam densitometer (Hologic, Inc., Bedford, Massachusetts). The instrumentation utilized in the laboratory inspection data and the remainder of the quality control details can be accessed on the NHANES website.^1^

### 2.2 Group definition

In accordance with the consensus published by ESPEN and EASO in 2022, and considering the diversity of racial types in the US population, we employed the diagnostic threshold values recommended in the consensus, based on NHANES data (6), which included low muscle mass (ALM/W, <0.257 for male; < 0.194 for female) (26) and a high fat mass percentage (FM%, >25 for male; >32% for female) (27). Individuals with SO characteristics were defined as a group when both criteria were met (S-O group), while the rest of the individuals were uniformly categorized (Non S-O group).

### 2.3 Assessment of TyG, TyG-WC, TyG-BMI, TyG-WCR, TyG-WHtR, TyG-WAR, HOMA-IR, BRI and LAP

The TyG Index is a quantitative measure of insulin resistance that is calculated by combining fasting blood glucose levels with triglyceride levels. Fasting blood glucose (FBG), triglycerides (TG), and insulin (IL) were assessed at the baseline stage of the study, when participants provided a blood sample. The following anthropometric measurements were obtained during the physical examination at the mobile screening center: weight, height, calf circumference (CC), arm circumference (AC), and waist circumference (WC). Additionally, the waist-calf circumference ratio (WCR), waist-height ratio (WHtR), waist-arm circumference ratio (WAR), HOMA-IR, BRI, and LAP were calculated using the following equations:


(1) $$TyG=\ln\left[TG\;\left(\mathrm{mg}/\mathrm{dl}\right)\times FBG\;\left(\mathrm{mg}/\mathrm{dl}\right)/2\right]$$

(2) $$WCR=\mathrm{Waist}\left(\mathrm{cm}\right)/\mathrm{Calf}-\mathrm{circumference}\left(\mathrm{cm}\right)$$

(3) $$\mathrm{WHtR}=\mathrm{Waist}\left(\mathrm{cm}\right)/\mathrm{Height}\left(\mathrm{cm}\right)$$

(4) $$\mathrm{WAR}=\mathrm{Waist}\left(\mathrm{cm}\right)/\mathrm{Arm}-\mathrm{circumference}\left(\mathrm{cm}\right)$$

(5) $$TyG-WC=TyG\times \mathrm{Waist}\left(\mathrm{cm}\right)$$

(6) $$TyG-BMI=TyG\times BMI\left(\mathrm{kg}/m^{2}\right)$$

(7) TyG−WCR=TyG×WCR

(8) TyG−WHtR=TyG×WHtR

(9) TyG−WAR=TyG×WAR

(10) $$\mathrm{HOMA}-IR=FPG\left(\mathrm{mg}/\mathrm{dl}\right)\times IL\left(\mu U/\mathrm{mL}\right)÷405$$

(11) $$BRI=364.2-365.5{\left[1-\pi^{-2}\times WC^{2}\left(m\right)\times \mathrm{Heigh}t^{-2}\left(m\right)\right]}^{1/2}$$

(12) $$\mathrm{Male}:\mathrm{LAP}=\left[WC\left(\mathrm{cm}\right)-65)\times TG\left(\mathrm{mmol}/L\right)\right]$$

(13) $$\mathrm{Female}:\mathrm{LAP}=\left[WC\left(\mathrm{cm}\right)-58)\times TG\left(\mathrm{mmol}/L\right)\right]$$

### 2.4 Assessment of covariates

Age and family income-to-poverty ratio (PIR) are treated as a continuous variable, gender is categorized into two groups (males and females), race/ethnicity is categorized into four segments (Mexican Americans, Non-Hispanic White people, Non-Hispanic Black people, and others), and education is categorized into three segments (less than high school, high school, and high school or above).

The participants were divided into two groups based on their smoking history: those who had smoked fewer than 100 cigarettes in their lifetime and those who had smoked more than 100 cigarettes. Alcohol consumption was defined as not drinking alcohol (fewer than 12 cups per year) and drinking alcohol (at least 12 cups per year). Self-reported hypertension and self-reported family history of diabetes were determined based on self-reported physician diagnosis obtained in a personal interview using a standardized medical status questionnaire. The participants were queried as follows: The participants were asked whether their physicians had informed them that they had high blood pressure or diabetes. They were instructed to respond with a yes or no answer.

Furthermore, LDL-cholesterol was quantified at baseline as a continuous variable, and total energy, protein, and total fat intake were calculated as the mean of the two-day intake when 2 days of dietary intake data were complete, and the first day’s intake otherwise.

The physical activity level was gaged with the Global Physical Activity Questionnaire and evaluated in accordance with the WHO guideline.^2^ Individuals who fulfilled the WHO physical activity recommendation were deemed to be physically active, which is defined as engaging in a minimum of 149 min of moderate physical activity, 74 min of vigorous physical activity, or 599 metabolic equivalent (MET) minutes per week. For more detailed information on covariate measurement, please refer to the NHANES website at (see text footnote 1, respectively).

### 2.5 Statistical analysis

In the correlation analysis for objective one of this study, statistical analysis for this segment incorporated sample weights due to the complex, multi-stage stratified probability survey design employed by NHANES, and was conducted in accordance with Centers for Disease Control and Prevention (CDC) guidelines.^3^ In the baseline characteristics table, continuous variables are presented as weighted means and standard errors (SE), while categorical variables are expressed as weighted proportions and standard errors (SE). For continuous variables, we employed weighted linear regression analysis to assess differences between participants with and without SO. For categorical variables, we utilized weighted chi-square tests for the same assessment. Subsequently, univariate and multivariate weighted logistic regression analyses were conducted to examine the relationships between TyG, TyG-WC, TyG-BMI, TyG-WCR, TyG-WHtR, TyG-WAR, HOMA-IR, BRI, LAP, and SO. Four models were applied in the study: Model 1 was unadjusted; Model 2 was adjusted for age, sex, race, marital status, and education level; Model 3 included additional adjustments for low-density lipoprotein on top of Model 2; Model 4 further adjusted for smoking, alcohol consumption, hypertension, diabetes, total energy intake, protein intake, and total fat intake on top of Model 3. In these four predefined models, the results are presented as odds ratios (OR) and 95% confidence intervals (CI). To elucidate the dose–response relationships between TyG, TyG-WC, TyG-BMI, TyG-WCR, TyG-WHtR, TyG-WAR, HOMA-IR, BRI, LAP, and SO, restricted cubic splines analysis (RCS) with the same covariates adjusted in Model 4 was conducted. Three knots were set to exclude the most extreme values, minimizing the potential impact of outliers, and likelihood ratio tests were used for nonlinearity testing, followed by flexible visualization.

In the second part of this study, focusing on the construction of a diagnostic model, we encountered a technical limitation: the R package ‘rms’ used does not support weighted processes. Consequently, during the model construction phase, we had to forgo the weighted variables. We randomly divided the dataset into a training set and a validation set in a 7:3 ratio. In the training set, we included 35 variables encompassing demographics, risk behaviors, anthropometrics, laboratory tests, and nutritional survey data. Initially, we identified potential predictive factors through univariate Logistic regression analysis. Variables were then selected based on a *p*-value less than 0.05 and included in the multivariate Logistic regression model. We employed the “backward” method for variable selection, ultimately identifying 16 variables. Furthermore, we utilized Lasso regression to select variables from a large set potentially subject to multicollinearity. This method determines the optimal penalty coefficient *λ* through 10-fold cross-validation. Ultimately, five key variables were selected: age, sex, WHtR, TyG-WCR, and BRI. Upon conducting a variance inflation factor (VIF) analysis for the model with these five variables, we found significant collinearity between BRI and WHtR (VIF > 10). Consequently, we constructed two 4-factor models, one including BRI and the other WHtR, and used the likelihood ratio test (LRT) to select the optimal model. The test results indicated no statistically significant difference between the two models. Subsequently, we conducted a difference significance test for the area under the receiver operating characteristic (ROC) curve using the Bootstrap method. It was found that the model including WHtR had a higher area under the ROC curve, and the difference was statistically significant. Considering the ease of obtaining WHtR in routine clinical practice, we selected age, sex, WHtR, and TyG-WCR as the variables for the final model. The VIF for the constructed model was less than 5, indicating no multicollinearity. Additionally, we used the Hosmer-Lemeshow test to assess the model’s goodness of fit, with a *p*-value greater than 0.05 indicating a well-fitted model. Next, we constructed dynamic nomograms and plotted the ROC curve, calibration curve, clinical decision curve (DCA), and clinical impact curve (CIC). For validation, we plotted using the same methods in the training set and compared the area under the ROC curves between the training and validation sets for significant differences. Additionally, we employed the Bootstrap method for internal validation, a statistical technique that simulates different datasets by randomly drawing samples repeatedly from the dataset (allowing the same sample to be chosen more than once) (28). We used 1,000 samples for this analysis to enhance the accuracy of the estimation. Lastly, we utilized random forest analysis to assess the relative importance of the four variables in model construction and visualized their contribution to the model.

Ultimately, physical activity will be incorporated as a covariate adjustment in one sensitivity analysis.

R software, version 4.1.3 (R Foundation, Vienna, Austria) was used to perform all statistical analyses. Correlation analyses were performed using the R software package “nhanesR,” Lasso regression was performed using the R software package “glmnet,” R software package “pROC” was used for the ROC analysis, and calibration curves were plotted using the R package “calibrate.,” DCA curve analysis using the R package “dca,” and image plotting using the “dplyr” package.

## 3 Results

### 3.1 Basic characteristics of participants according to the SO

This study included a total of 5,574 American adults, of whom 323 had SO and 5,251 did not. Baseline characteristic analysis revealed statistically significant differences between the two groups for multiple variables, including the TyG.(for details, see Supplementary Table S1).

### 3.2 Relationship between TyG, TyG-WC, TyG-BMI, TyG-WCR, TyG-WHtR, TyG-WAR, HOMA-IR, BRI and LAP and SO

After adjusting for all covariates in the multivariable Logistics regression, the associations between TyG, TyG-WC, TyG-BMI, TyG-WCR, TyG-WHtR, TyG-WAR, HOMA-IR, BRI, LAP, and SO were all statistically significant and positively correlated. Among patients with SO, the association with TyG-WHtR was the strongest (OR: 4.01, 95% CI: 3.21–5.00), followed by TyG-WC (OR: 2.12, 95% CI: 1.87–2.41), BRI (OR: 1.81, 95% CI: 1.65–1.99), and TyG (OR: 1.81, 95% CI: 1.36–2.42). The results are shown in Table 1.

**Table 1:** Relationship between insulin resistance-related indicators and obesity-related indicators and S-O.

Characteristic	OR (95% CI)	*p*	OR (95% CI)	*p*	OR (95% CI)	*p*	OR (95% CI)	*p*
S-O (ESPEN&EASO)								
	Model 1		Model 2		Model 3		Model 4	
TyG	2.74 (2.22,3.39)	<0.0001	1.93 (1.51,2.47)	<0.0001	1.94 (1.53,2.46)	<0.0001	1.81 (1.36,2.42)	<0.001
TyG-WCR	1.47 (1.39,1.56)	<0.0001	1.43 (1.34,1.54)	<0.0001	1.43 (1.33,1.53)	<0.0001	1.44 (1.35,1.54)	<0.0001
TyG-WHtR	3.37 (2.89,3.92)	<0.0001	3.77 (3.13,4.53)	<0.0001	3.70 (3.08,4.44)	<0.0001	4.01 (3.21,5.00)	<0.0001
TyG-WAR	1.31 (1.26,1.36)	<0.0001	1.34 (1.28,1.40)	<0.0001	1.33 (1.27,1.40)	<0.0001	1.35 (1.28,1.43)	<0.0001
TyG-BMI	1.01 (1.01,1.02)	<0.0001	1.02 (1.01,1.02)	<0.0001	1.02 (1.01,1.02)	<0.0001	1.02 (1.01,1.02)	<0.0001
TyG-WC	1.94 (1.79,2.11)	<0.0001	2.05 (1.85,2.27)	<0.0001	2.03 (1.83,2.25)	<0.0001	2.12 (1.87,2.41)	<0.0001
BRI	1.62 (1.53,1.72)	<0.0001	1.80 (1.66,1.95)	<0.0001	1.79 (1.65,1.94)	<0.0001	1.81 (1.65,1.99)	<0.0001
HOMA-IR	1.13 (1.09,1.17)	<0.0001	1.14 (1.10,1.18)	<0.0001	1.14 (1.10,1.18)	<0.0001	1.12 (1.07,1.17)	<0.0001
LAP	1.01 (1.01,1.02)	<0.0001	1.01 (1.01,1.02)	<0.0001	1.01 (1.01,1.02)	<0.0001	1.01 (1.01,1.02)	<0.0001

Model 1 was unadusted. Model 2 was adjusted for age, gender, ethnicity, marriage and education. Model 3 was adjusted for age, gender, ethnicity, marriage, education and LDL. Model 4 was adjusted for age, gender, ethnicity, marriage, education, LDL, smoke, drink, hyperyension, diabetes, energy-intake, protein-intake, total fat-intake. S-O, Individuals with body composition characteristics of sarcopenic obesity; ESPEN&EASO, The European Society for Clinical Nutrition and Metabolism and the European Association for the Study of Obesity; WCR, Waist-to-calf circumstance ratio; WHtR, Waist-to-height circumstance ratio; WAR, Waist-to-arm circumstance ratio; BMI, Body Mass Index; WC, Waist Circumference; TyG, Triglyceride-Glucose index; BRI, body roundness index; HOMA-IR, Homeostatic Model Assessment for Insulin Resistance; LAP, Lipid Accumulation Product.

### 3.3 Restricted cubic splines analysis investigating the relationship between TyG, TyG-WC, TyG-BMI, TyG-WCR, TyG-WHtR, TyG-WAR, HOMA-IR, BRI and LAP and S-O

**Figure 1:**
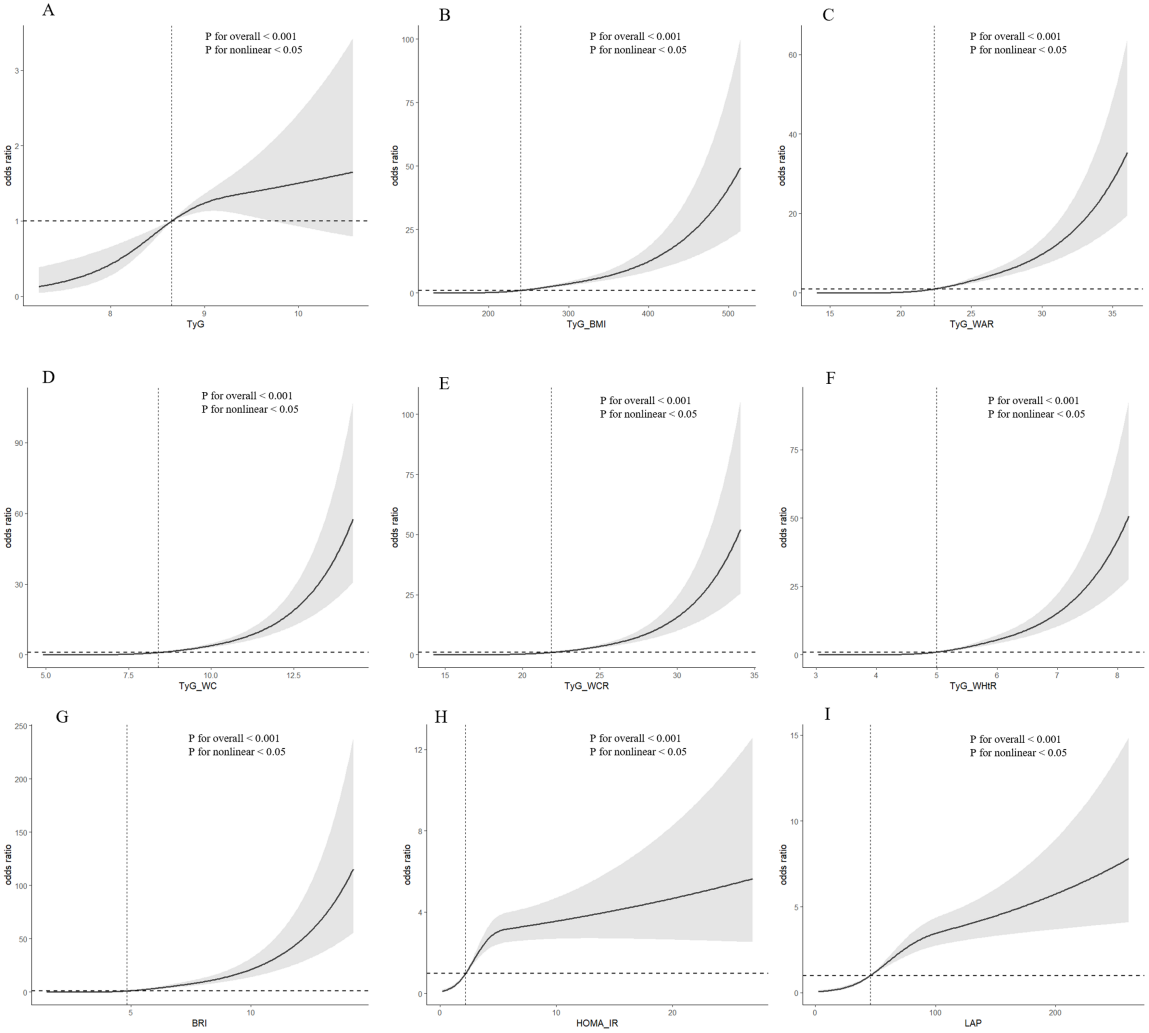
**(A)** RCS analysis results for TyG and SO; **(B)** RCS analysis results for TyG-BMI and SO; **(C)** RCS analysis results for TyG-WAR and SO; **(D)** RCS analysis results for TyG-WC and SO; **(E)** RCS analysis results for TyG-WCR and SO; **(F)** RCS analysis results for TyG-WHtR and SO; **(G)** RCS analysis results for BRI and SO RCS analysis results of SO; **(H)** RCS analysis results of HOMA-IR and SO; **(I)** RCS analysis results of LAP and SO.

In Figure 1, we utilized RCS visualization to depict the nonlinear relationships between independent and dependent variables. After adjusting for all covariates in the aforementioned Model 4, we found that the nonlinear relationships between TyG, TyG-WC, TyG-BMI, TyG-WCR, TyG-WHtR, TyG-WAR, HOMA-IR, BRI, LAP, and SO were all statistically significant (*p* values for all <0.001; p values for nonlinearity <0.05).

### 3.4 Binary logistic regression analysis combined with Lasso regression analysis to screen predictor variables

**Figure 2:**
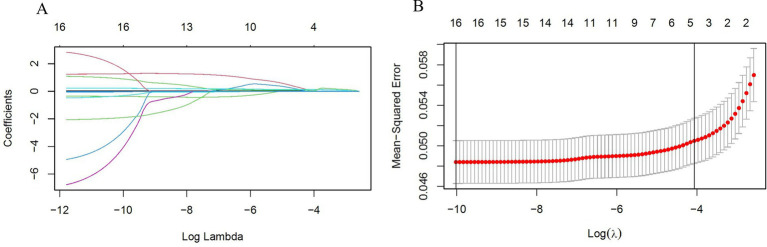
**(A)** LASSO selection path diagram: the vertical dashed line on the left side of the diagram indicates Log(λ) corresponding to the minimum error (lambda.1se), while the vertical dashed line on the right side of the diagram indicates that Log(λ) differs from the minimum error (lambda.min) by one standard error; **(B)** LASSO path diagram: curve of regression coefficients versus Log(λ) as the coefficient scores gradually decrease.

The statistical analysis procedures have been previously detailed in the statistical methods section. The results of the univariate and multivariate Logistic regression analyses are presented in Table 2. Following univariate analysis, Logistic regression was performed to distill the data, identifying 16 variables. Subsequently, Lasso regression was conducted with the addition of a penalty coefficient, followed by 10-fold cross-validation, as shown in Figure 2. Utilizing one standard error of the mean as the threshold, we selected the number of variables represented by the rightmost dashed line, ultimately identifying five variables. Due to collinearity between BRI and WHtR, we elected to retain WHtR for model construction, and the Hosmer-Lemeshow test resulted in a significance of 0.497, indicating good model fit within the training set.

**Table 2:** The results of the risk factor discovery model.

Characteristics	Univariate analysis			Multivariate analysis		
	OR	95% CI	*p*	OR	95% CI	*p*
Age	1.05	1.04–1.06	<0.001	1.04	1.03–1.05	<0.001
PIR	0.88	0.81–0.96	0.003			
Weight	1.03	1.02–1.04	<0.001	0.91	0.84–0.99	0.038
BMI	1.11	1.10–1.13	<0.001			
WC	1.07	1.06–1.08	<0.001			
CC	1.07	1.04–1.10	<0.001			
AC	1.06	1.01–1.11	0.02	0.56	0.36–0.87	0.01
FBG	1.01	1.01–1.01	<0.001			
TG	1.01	1.00–1.01	<0.001	1.01	1–1.02	0.073
LDL	1.00	0.99–1.00	0.02	0.99	0.99–1	0.015
IL	1.03	1.02–1.04	<0.001			
Energy-intake (kcal)	1.00	1.00–1.00	<0.001			
Protein-intake (gm)	0.99	0.99–0.99	<0.001	0.99	0.99–1.00	0.004
Total fat-intake (gm)	0.99	0.99–1	0.001			
wBMI (kg/m)	1.07	1.06–1.08	<0.001			
WCR	110.32	64.61–188.39	<0.001			
WHtR	1.13	1.12–1.15	<0.001	3.34	2.25–4.96	<0.001
WAR	17.81	12.57–25.25	<0.001	0.00	0.00–0.53	0.029
TyG	2.55	2.05–3.17	<0.001			
TyG-WCR	1.43	1.37–1.5	<0.001	1.26	1.14–1.4	<0.001
TyG-WHtR	3.37	2.92–3.89	<0.001	0.00	0.00–0.03	0.001
TyG-WAR	1.31	1.27–1.36	<0.001			
TyG-BMI	1.01	1.01–1.01	<0.001	1.03	1.00–1.06	0.029
TyG-WC	1.95	1.79–2.11	<0.001	37.06	3.56–385.59	0.003
BRI	1.6	1.52–1.69	<0.001	0.12	0.05–0.29	<0.001
HOMA-IR	1.09	1.06–1.12	<0.001			
LAP	1.01	1.01–1.02	<0.001	0.99	0.97–1	0.068
Sex	1.73	1.32–2.27	<0.001	3.43	2.11–5.59	<0.001
Ethnicity	0.89	0.77–1.02	0.104			
Marriage	0.98	0.78–1.22	0.833			
Education	0.83	0.71–0.98	0.023			
Smoke	1.19	0.91–1.54	0.203			
Drink	0.81	0.61–1.07	0.138			
Hypertension	3.19	2.44–4.17	<0.001			
Diabetes	1.03	1.02–1.04	<0.001	0.71	0.44–1.13	0.153

S-O, Individuals with body composition characteristics; PIR, Family income-to-poverty ratio; DXA, Dual-energy X-rays Absorptiometry; FM%, Fat mass%; BMI, Body Mass Index; WC, Wasit Circumference; CC, Calf Circumference; ALM, Appendicular Lean Mass; wBMI, Waist Body Mass Index; WCR, Waist-to-Calf Circumstance Ratio; WHtR, Waist-to-Height Ratio; WAR, Waist-to-Arm Ratio; TyG, Triglyceride-Glucose index; BRI, body roundness index; HOMA-IR, Homeostatic Model Assessment for Insulin Resistance; LAP, Lipid Accumulation Product.

### 3.5 Dynamic nomogram development and validation

**Figure 3:**
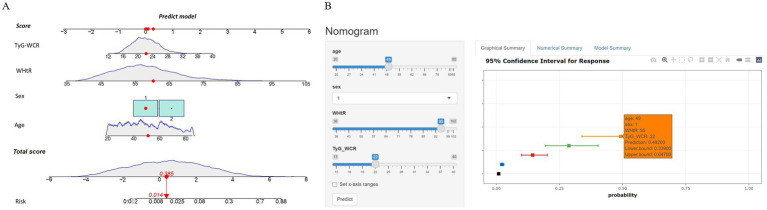
**(A)** Interactive nomogram; **(B)** Dynamic nomogram.

**Figure 4:**
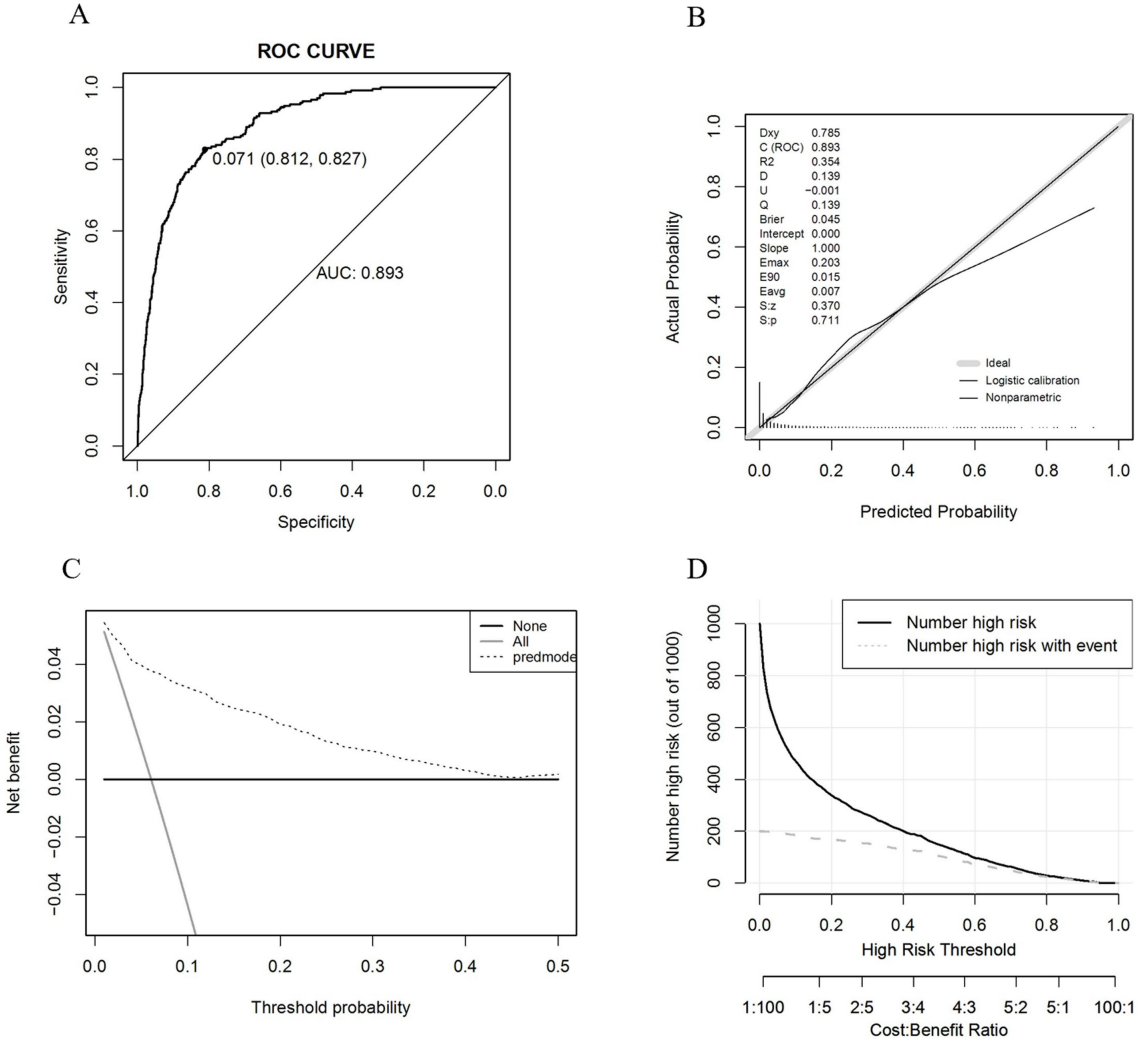
**(A)** ROC graph; **(B)** Calibration graph; **(C)** DCA graph; **(D)** CIC graph.

After finalizing the variables for the sarcopenic obesity diagnostic model in American adults (Figure 3), we constructed the model using the ‘rms’ package in RStudio and plotted interactive nomograms with the ‘regplot’ package. We developed dynamic nomograms with the ‘DynNom’ package, which were saved using the ‘DNbuilder’ package (for details, see Supplementary materials, DynNomApp). To further evaluate the predictive ability of the model, we plotted the ROC curve, calibration curve, DCA curve, and CIC curve, as shown in Figure 4. The area under the ROC curve (AUC) was 0.897, which was higher than that of the variables included in the model: TyG-WCR (AUC = 0.842), WHtR (AUC = 0.842), age (AUC = 0.742), and sex (AUC = 0.567). The calibration curve showed a Brier score of 0.045, indicating good predictive performance of the model, with an average absolute error of 0.005 for the mean calibration plot.

**Figure 5:**
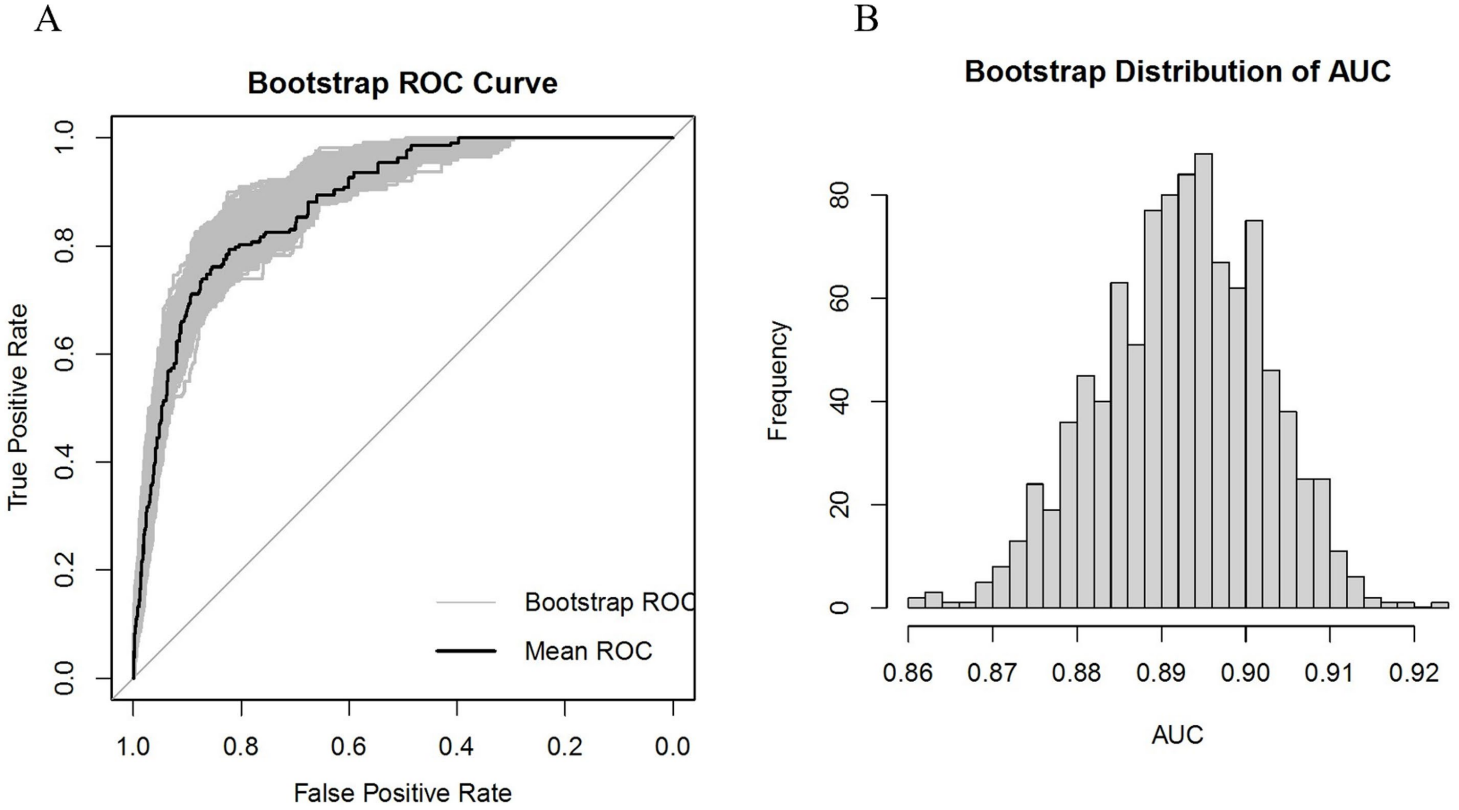
**(A)** Bootstrap-based ROC graph; **(B)** AUC distribution graph based on Bootstrap method.

Subsequently, internal validation was conducted using the Bootstrap method, and the ROC curve along with the AUC frequency distribution plot were depicted, as shown in Figure 5. The calibration and DCA curves are presented in the Supplementary Figure S2, for details. The results indicate that the model’s AUC is well-distributed, ranging from 87.4 to 91.1%, while the Brier score ranges from 4.0 to 5.0%.

**Figure 6:**
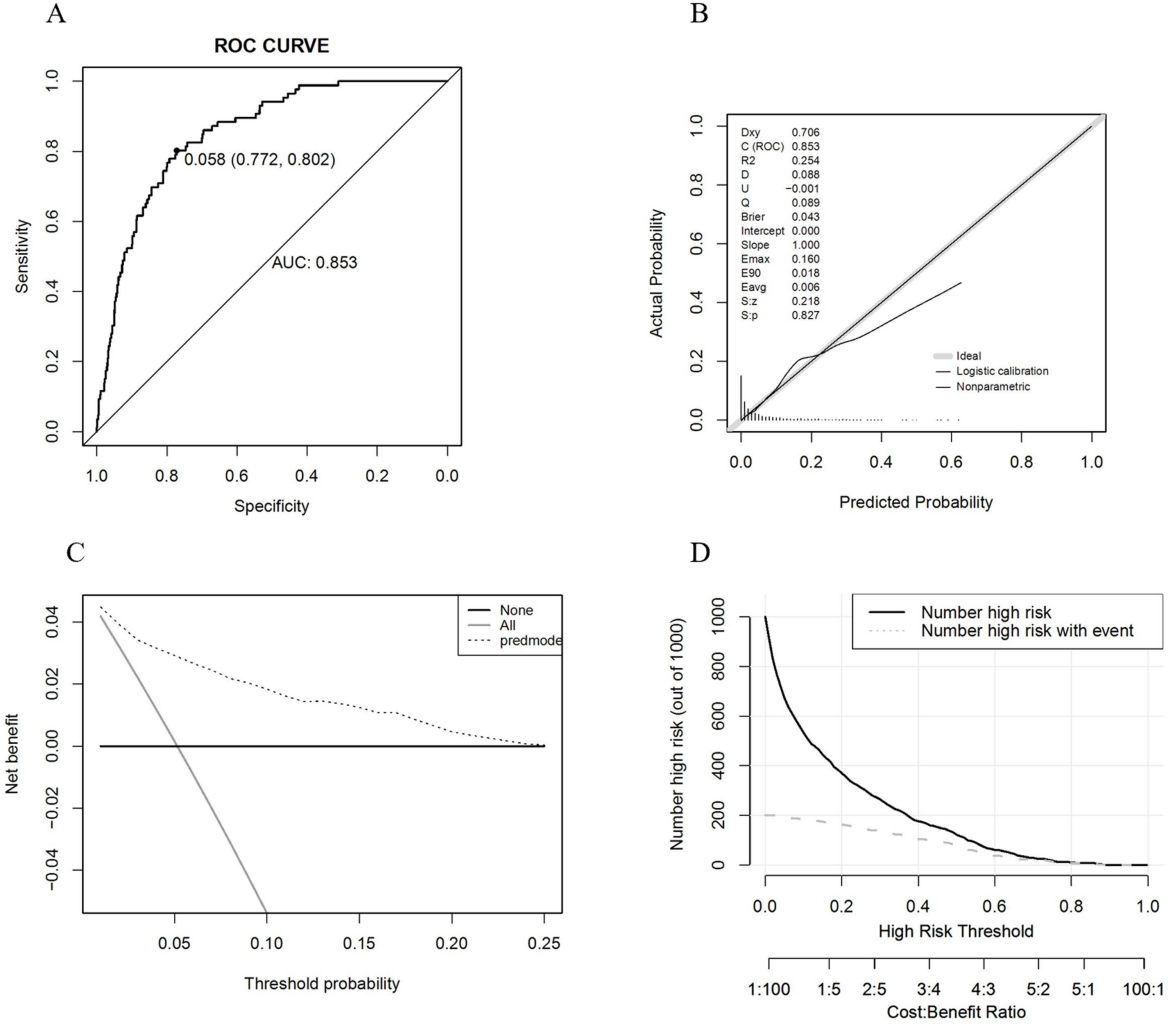
**(A)** ROC graph; **(B)** Calibration graph; **(C)** DCA graph; **(D)** CIC graph.

We proceeded with the validation using the validation set and the results are shown in Figure 6. The AUC was 0.853, and the calibration curve indicated a Brier score of 0.043, suggesting good predictive performance of the model. The mean calibration plot showed an average absolute error of 0.006. Additionally, we utilized the ‘roc.test’ package to assess the AUC, yielding a *p*-value of 0.068, which suggests no significant difference between the training and validation datasets. The Hosmer-Lemeshow test showed a significance level of 0.925, further confirming the model’s good fit.

**Figure 7:**
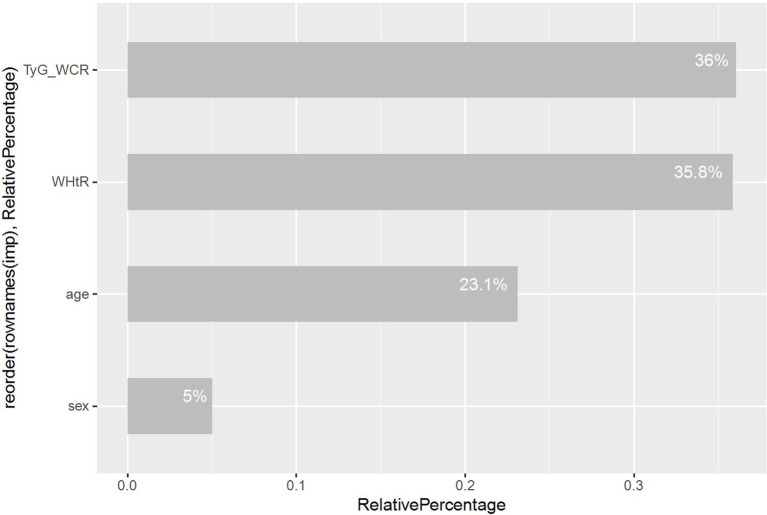
Relative importance of machine learning (random forest) outputs.

Ultimately, we utilized random forest analysis to determine the relative importance of variables, providing a clearer view of each variable’s contribution within the model, as depicted in Figure 7.

### 3.6 Sensitivity analysis

A sensitivity analysis was conducted to examine the impact of physical activity on the study outcomes. Furthermore, data pertaining to physical activity were extracted from the primary analytic dataset and incorporated as a continuous variable in multifactorial regression models. A total of 4,150 Americans were included in the study results, of whom 212 were diagnosed with SO. Univariate analyses demonstrated no statistical significance at *p* = 0.23 (see T2 for details). It was therefore prudent to include them in a multifactorial regression. The results demonstrated that, following adequate adjustment for covariates, the findings remained consistent with those of the main analysis (see T3 for details). As the one-way analysis was not statistically significant, it could not be included in the model construction. Consequently, the results of the sensitivity analysis indicate that the conclusions drawn from this study are stable and reliable.

## 4 Discussion

Our study, for the first time, explores the correlation between insulin resistance and obesity indicators with SO patients, building upon existing literature. Utilizing RCS analysis, we intuitively demonstrated the nonlinear relationships between these variables. The study found that as the TyG increases, the risk of SO shows a progressively increasing trend. However, when we multiply TyG by other obesity-related indicators (WC, BMI, WCR, WHtR, WAR) to form composite indicators, it is found that these composite indicators have a significantly positive correlation with the risk of SO. This positive correlation may stem from the composite indicators’ consideration of body morphology along with corresponding adjustments, thereby endowing the TyG composite indicators with superior predictive power compared to the standalone TyG indicator. This finding is consistent with previous research outcomes (22).

As an effective measure of obesity, the significant positive correlation between an increase in BRI and the risk of SO persists after adjustment for all covariates. On the other hand, as an effective indicator of insulin resistance, the association between HOMA-IR and SO in the fully adjusted model (Model 4) shows a lower odds ratio compared to TyG and its composite indicators, suggesting that TyG, as a rapid and convenient measure of insulin resistance, may have superior predictive power to HOMA-IR, a finding that has been confirmed in previous research (21). Concurrently, another obesity indicator—LAP shows a lower odds ratio in Model 4 compared to BRI, and its nonlinear relationship with SO exhibits a more modest upward trend, which may imply that LAP’s performance in predicting SO is not as strong as that of BRI.

We constructed a nomogram to visually represent our diagnostic model, highlighting that advanced age, male, high WHtR, and high TyG-WCR are significant risk factors for sarcopenic obesity in the United States population. Consensus published in 2022 on sarcopenic obesity suggests that obese and overweight individuals over the age of 70 should be considered at risk for SO, as previous studies have shown a significant increase in the prevalence of sarcopenia with advancing age (29). Epidemiological data indicate a rising incidence of SO in older adults, with those aged 65 and above being at a higher risk (8). Prior research has linked the development of sarcopenic obesity to the crosstalk between adipose and skeletal muscle tissues associated with aging, which is considered one of the primary mechanisms (16). The accumulation of adipose tissue increases intramuscular fat infiltration, leading to an increase in intermuscular free fatty acids and a dysregulation of adipokines (30), particularly the shift from adiponectin to leptin/Monocyte chemoattractant protein-1 (MCP-1), also known as Chemokine ligand 2 (CCL2) (31). Alongside other adipokines, they can bind to CC chemokine receptor 2 (CCR2), cluster of differentiation 36 (CD36), and/or toll-like receptor 4 (32), further influencing the macrophage phenotype from an anti-inflammatory (M2) to a pro-inflammatory (M1) state, M2 macrophages, predominant in leaner individuals, play a role in improving glucose metabolism and maintaining insulin sensitivity, yet their specific efficacy depends on Transforming Growth Factor-beta (TGF-*β*) (33). Adipose tissue not only promotes the activation of pro-inflammatory Type 1 helper T cells (Th1) but also induces a shift in T lymphocyte types from the anti-inflammatory Th2 and Treg types to the pro-inflammatory Th1 and Th17 types. This transition is accompanied by the secretion of interferon-*γ*, further activating M1 macrophages (34). In this pro-inflammatory environment, adipokines are activated, which not only induce muscle cell apoptosis but also affect the regulatory mechanisms of muscle atrophy activation proteins (16). Studies indicate that the transition between M1 and M2 macrophages is closely related to the development of systemic IR (35). These findings provide theoretical support for our model, explaining the choice of TyG-WCR, adjusted for WCR, as an indicator of insulin resistance when applying Lasso regression analysis to address multicollinearity in composite indicators. This indicator, proposed in this study, takes into account the impact of WCR on insulin resistance, potentially offering a more accurate predictive tool. The study results show that as TyG-WCR increases, the risk of SO rises significantly, and its predictive power surpasses that of the standalone TyG indicator. This advantage may stem from WCR being a composite indicator of WC and CC, which not only considers early screening for central obesity (using WC as an indicator) (36), but also early screening for sarcopenia (using CC as an indicator) (37), thereby providing a reasonable explanation for our results. A high WHtR is considered a risk factor for SO in our study, consistent with previous research findings (38). The random forest algorithm, when assessing the relative importance of variables in the model, indicates that gender is not a key predictive factor. Nonetheless, the consideration of gender in our diagnostic model may provide value for gender-specific predictions. This is because the diagnostic criteria for SO set different thresholds for the same variables between genders, highlighting the importance of gender specificity in diagnostic and predictive models (6).

The robustness of the model was ensured through the scientific identification of key risk factors and the treatment of covariates. The predictive performance of the model was satisfactory when evaluated internally and in the validation set; however, further external validation of the model is necessary in the future. In addition, the variables included in the model are easily accessible basic indicators, which makes the model a potentially effective diagnostic model for sarcopenic obesity, facilitating future screening for the disease in parts of the country where instrumentation limitations exist.

It is essential to acknowledge the constraints of this study. Firstly, the cross-sectional data utilized in this study were employed solely for the construction of the diagnostic model. In the future, it is imperative to confirm and further develop our model using cohort studies for these variables in predicting disease occurrence. Secondly, the nomogram has only been internally validated; thus, further external validation is necessary to ensure its reliability and generalizability. It should be noted, however, that NHANES is a cross-sectional national survey conducted annually. Consequently, it would be beneficial to validate our model using the upcoming NHANES dataset in future studies. Third, it should be recognized that our dataset only contains information on the U.S. population. However, the U.S. is a multiracial, cosmopolitan country and a country of immigrants with multicultural backgrounds, which may result in the representation of different racial groups.

Nonetheless, our study reveals the association between insulin resistance and obesity indicators with SO and visualizes this relationship, laying a solid foundation for future research. We anticipate that our model will receive broader external validation in the future, to facilitate the development of early screening and prevention efforts for SO.

## 5 Conclusion

After adjusting for potential confounding factors in the US population, our study found that the TyG, as well as combinations of TyG with other obesity indicators (TyG-WC, TyG-BMI, TyG-WCR, TyG-WHtR, TyG-WAR), HOMA-IR, BRI, and LAP, were all significantly associated with SO. It is therefore recommended that these indicators be given full consideration in future studies, with a view to deepening our understanding of the risk factors associated with SO.

The utilization of nomograms or dynamic nomograms in diagnostic models by clinical staff enables the rapid identification of individuals at risk of SO. This ability to rapidly identify is of critical importance for enabling the early detection, intervention and treatment of SO. Furthermore, our study, based on a nationally representative dataset, highlights that the U.S. population of advanced age, males with high WHtR, and individuals with high TyG-WCR are key targets for focused attention and preventive measures.

## Data Availability

The original contributions presented in the study are included in the article/Supplementary material, further inquiries can be directed to the corresponding author/s.

## Footnotes

^1^https://www.cdc.gov/nchs/nhanes/index.htm

^2^https://www.who.int/ncds/surveillance/steps/resources/GPAQ_Analysis_Guide.pdf

^3^https://wwwn.cdc.gov/nchs/nhanes/tutorials/default.aspx
