# Supplementary figures and images for: Correlation of insulin resistance-related indicators and obesity-related indicators with sarcopenic obesity and development of diagnostic models: NHANES 1999–2006
Source: Front Nutr. 2024 Nov 28;11:1492191. doi: 10.3389/fnut.2024.1492191 (PMC11635994; doi:10.3389/fnut.2024.1492191)

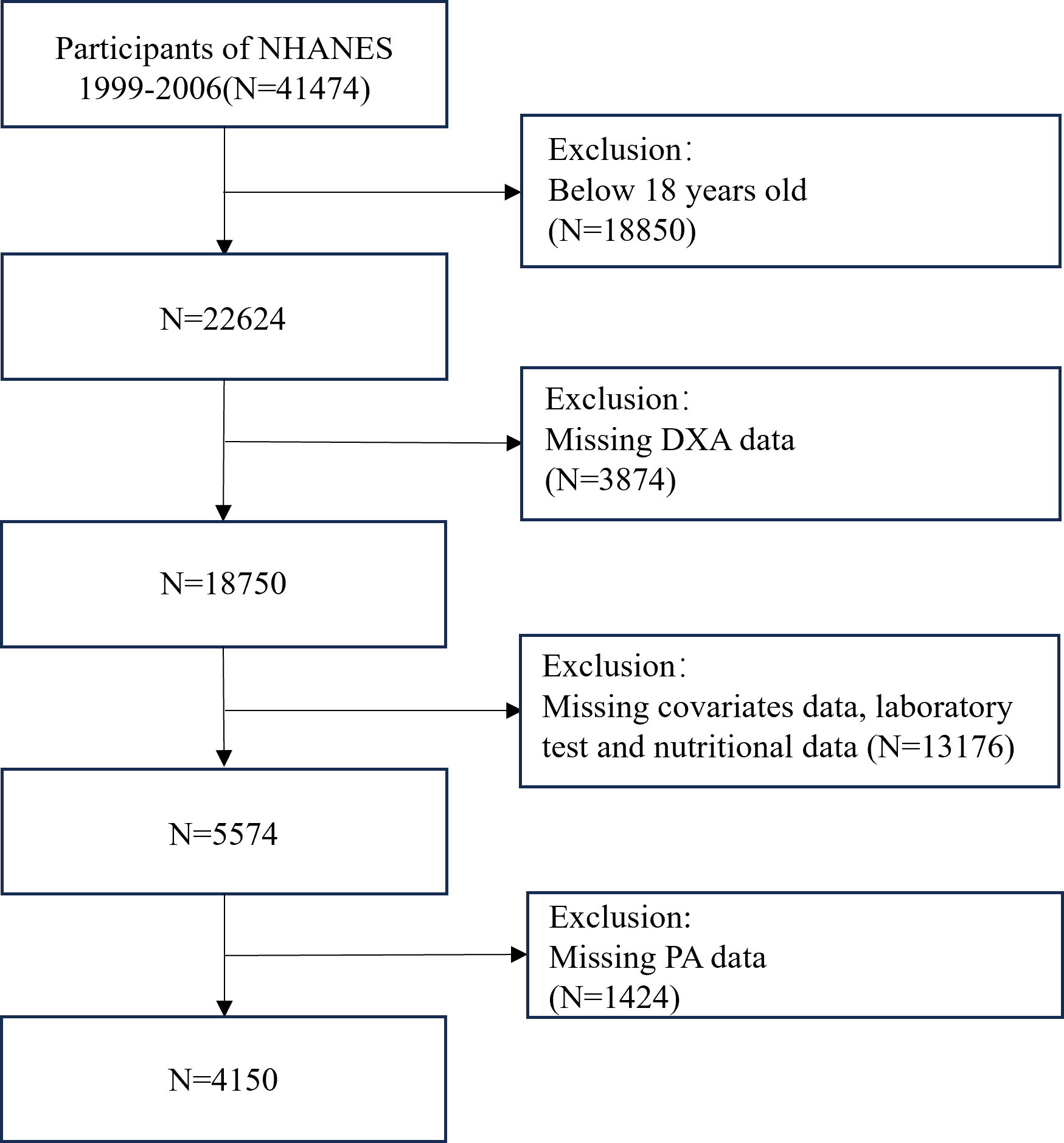

Supplement: Supplementary file 1 [file Data_Sheet_1.ZIP › Supplementary Material/F1.tif]

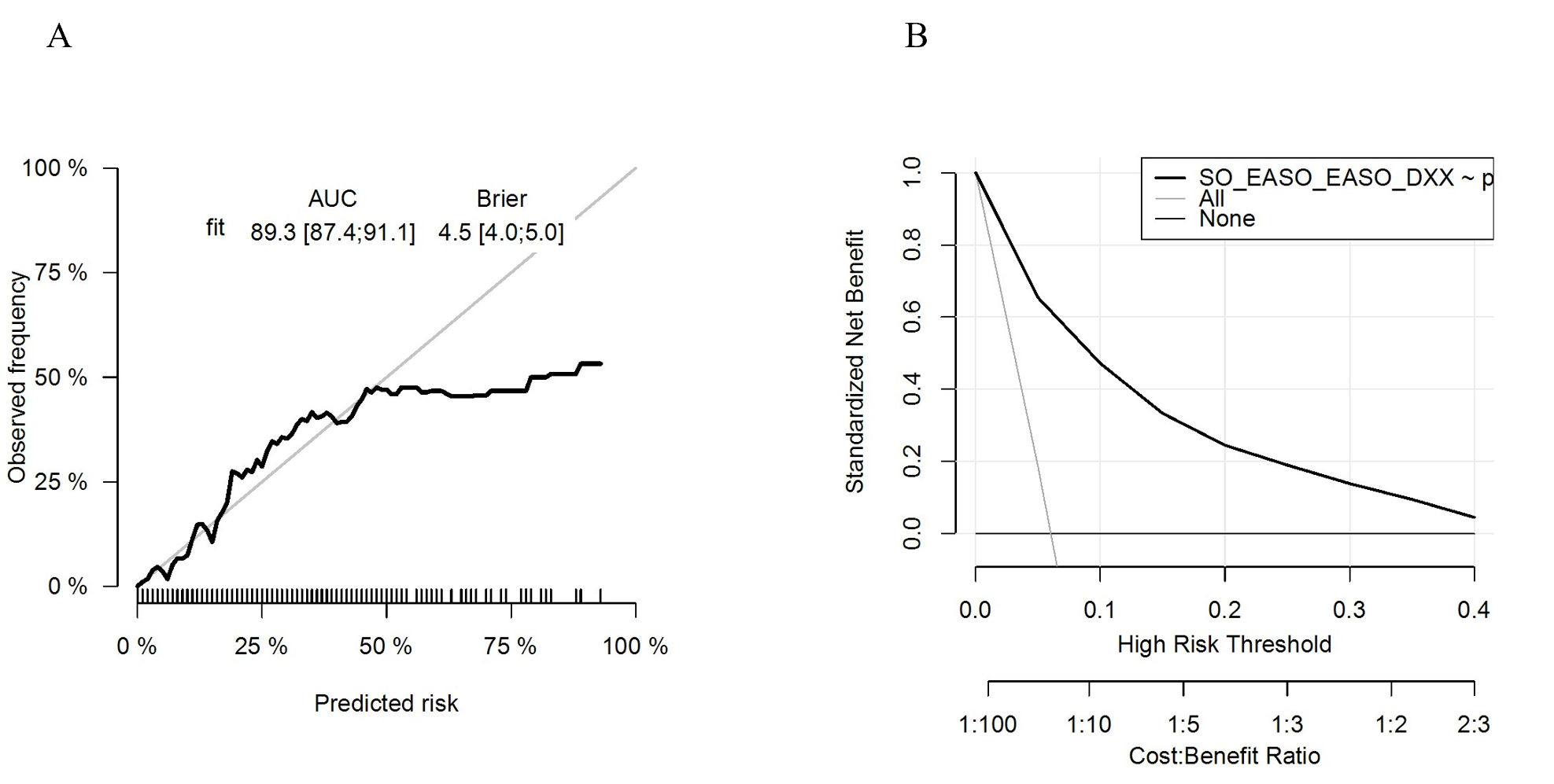

Supplement: Supplementary file 1 [file Data_Sheet_1.ZIP › Supplementary Material/F2.tif]
